# A sudden rise in pacing threshold of left ventricular lead associated with myocardial ischemia

**DOI:** 10.1002/joa3.12536

**Published:** 2021-04-03

**Authors:** Yusuke Tomita, Shinya Yamada, Takashi Kaneshiro, Naoko Hijioka, Takeshi Shimizu, Yasuchika Takeishi

**Affiliations:** ^1^ Department of Cardiovascular Medicine Fukushima Medical University Fukushima Japan; ^2^ Department of Arrhythmia and Cardiac Pacing Fukushima Medical University Fukushima Japan

## Abstract

We report for the first time a sudden rise in the pacing threshold of the left ventricular lead due to myocardial ischemia after cardiac resynchronization therapy with defibrillator implantation, and its recovery to the baseline after the revascularization.

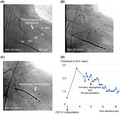

## 1 INTRODUCTION

Myocardial ischemia may become a cause of increases in pacing lead threshold after implantation of a cardiovascular implantable electronic device. Acute changes in the right ventricular (RV) and atrial lead pacing threshold following myocardial infarction have been demonstrated. Here, we report for the first time a sudden rise in the pacing threshold of the left ventricular (LV) lead because of myocardial ischemia after cardiac resynchronization therapy with defibrillator (CRT‐D) implantation, and its recovery to the baseline after revascularization.

## 2 CASE REPORT

**FIGURE 1:**
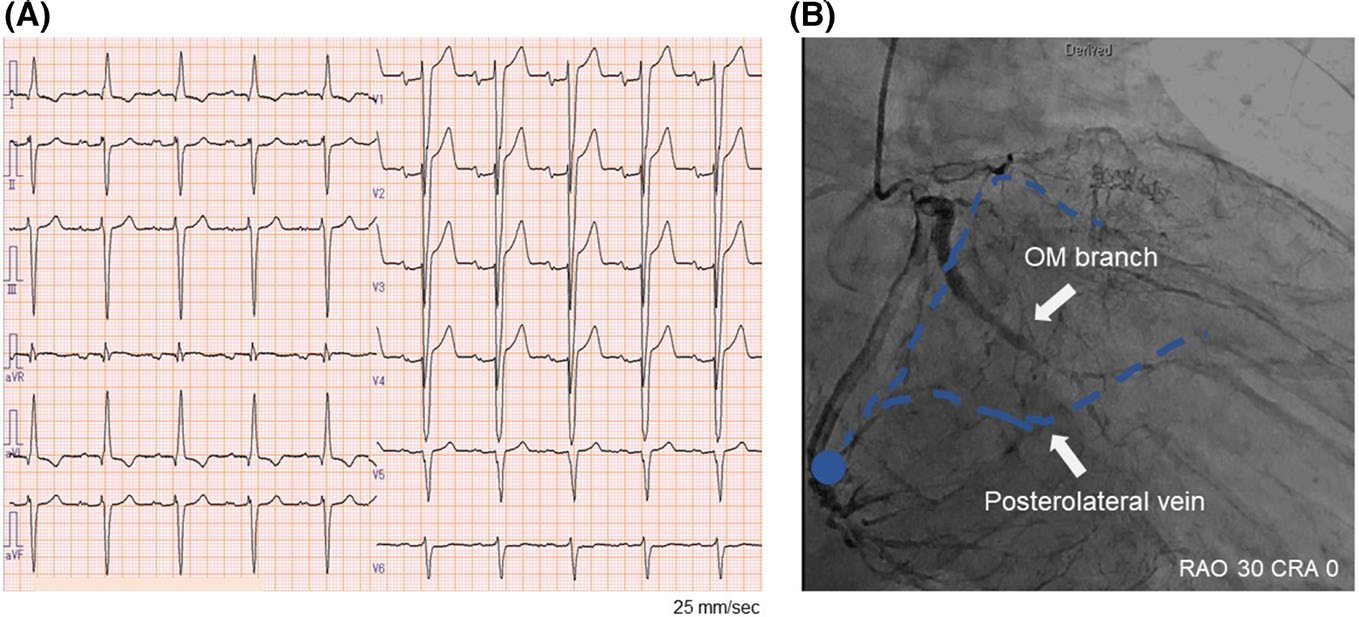
Electrocardiogram and coronary angiography before cardiac resynchronization therapy with defibrillator (CRT‐D) implantation. A, Electrocardiogram before CRT‐D implantation. B, Coronary angiography 7 d before CRT‐D implantation. The blue dotted lines indicate the coronary venous route. CRA, cranial; OM, obtuse marginal; RAO, right anterior oblique

**FIGURE 2:**
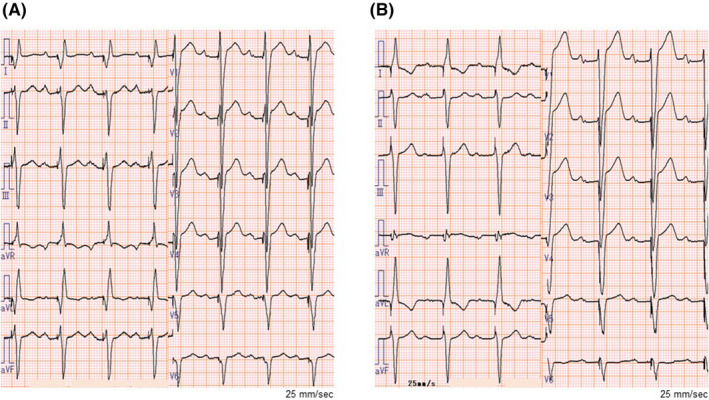
Electrocardiogram after cardiac resynchronization therapy with defibrillator (CRT‐D) implantation. A, Biventricular pacing just after CRT‐D implantation. B, Left ventricular lead pacing failure 4 d after CRT‐D implantation

**FIGURE 3:**
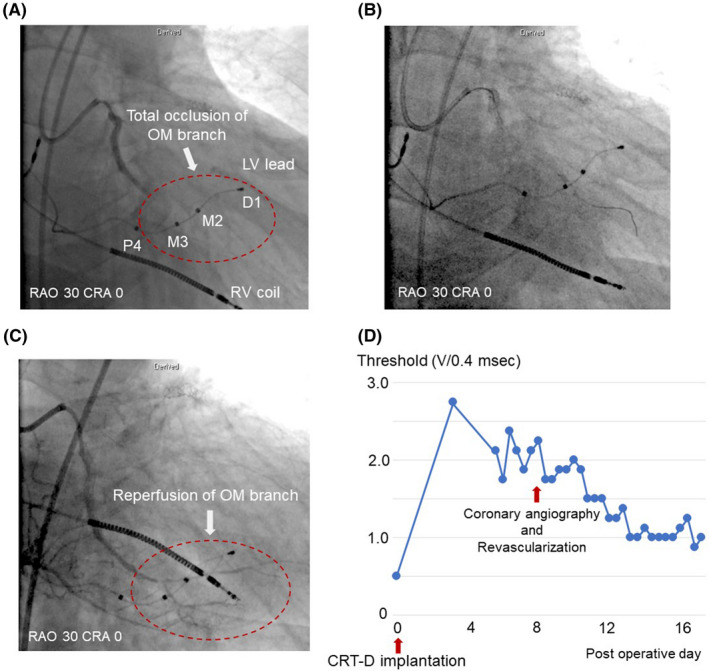
Coronary angiography (A‐C) and changes in left ventricular pacing threshold (D). A, Coronary angiography 8 d after CRT‐D implantation. The red dotted circle indicates the regions perfused by the OM branch. B, Revascularization of the OM branch. C, Improved perfusion of the OM branch around the LV pacing lead electrodes. D, Changes in the LV pacing lead threshold (Mid 2–RV coil vector) after CRT‐D implantation. CRA, cranial; CRT‐D, cardiac resynchronization therapy with defibrillator; D1, Distal 1; LV, left ventricular; M2, Mid 2; M3, Mid 3; OM, obtuse marginal; P4, Proximal 4; RAO, right anterior oblique; RV, right ventricular

A 73‐year‐old man suffered symptomatic heart failure with the left ventricular (LV) ejection fraction of 28% and intraventricular conduction disturbance (QRS duration 170 ms, Figure 1A) because of broad anterior wall myocardial infarction diagnosed 24 years previously. He had a past history of diabetes. Elective implantation of CRT‐D (Quadra Assura MP™, Abbott) was performed. In the present case, clopidogrel was used after a prior myocardial infarction. The use of clopidogrel (75 mg/d) was discontinued 2 days before CRT‐D implantation and was resumed 4 days after CRT‐D implantation. The LV pacing lead was positioned in the posterolateral vein because of preserved coronary artery blood flow in the targeted area observed on coronary angiography 7 days before CRT‐D implantation (Figure 1B). At baseline, LV capture was found at the multiple pacing vectors (Table 1). However, electrocardiographic changes from biventricular pacing (Figure 2A) to LV lead pacing failure (Figure 2B) were seen on the 12‐lead electrocardiogram 4 days after CRT‐D implantation. Subsequent device check revealed that a sudden rise in the pacing threshold occurred at the multiple pacing vectors. Dislocation of the LV lead was not detected by fluoroscopy. Automatic ventricular threshold measurement was performed every 8 hours after a sudden rise in the pacing threshold, and the threshold of the LV pacing lead remained high until coronary angiography was performed 8 days after CRT‐D implantation. Surprisingly, coronary angiography revealed a total occlusion of the obtuse marginal (OM) branch that perfused the area around the LV lead electrodes (Figure 3A), and we immediately performed plain old balloon angioplasty for the occluded OM branch (Figure 3B,C). The pacing threshold of the LV lead (Mid 2–RV coil vector) was 0.5 V/0.4 ms at baseline and 2.75 V/0.4 ms 4 days after CRT‐D implantation. However, after revascularization of the OM branch, the pacing threshold was gradually decreased and recovered to the baseline level 5 days after revascularization (Figure 3D).

**TABLE 1:** Acute changes in left ventricular pacing lead threshold after cardiac resynchronization therapy with defibrillator implantation

Pacing vector	Baseline	4 d after
Distal 1–Mid 2	5.0 V/0.4 ms	>5.0 V/0.4 ms
Distal 1–Proximal 4	2.2 V/0.4 ms	>5.0 V/0.4 ms
Mid 2–Proximal 4	0.5 V/0.4 ms	>5.0 V/0.4 ms
Mid 3–Proximal 4	>5.0 V/0.4 ms	>5.0 V/0.4 ms
Mid 2–Right ventricular coil	0.5 V/0.4 ms	2.75 V/0.4 ms
Mid 3–Right ventricular coil	>5.0 V/0.4 ms	>5.0 V/0.4 ms
Proximal 4–Right ventricular coil	1.7 V/0.4 ms	>5.0 V/0.4 ms

## 3 DISCUSSION

A quadripolar LV pacing lead enables to overcome a high pacing threshold after device implantation by means of a range of programmed configurations. However, acute changes in the pacing lead threshold can occur following device implantation because of lead displacement and myocardial tissue inflammation just after lead positioning. Also, an acute rise in the pacing threshold following myocardial infarction has been reported after RV or right atrial lead positioning. In our case, lead displacement was excluded using fluoroscopy. In addition, changes in pacing lead threshold attributed to myocardial tissue inflammation might have been less likely because LV pacing lead was positioned in the venous system, and the influence of lead positioning on myocardial tissue could be small. Therefore, myocardial ischemia was considered as the main cause of a sudden rise in the LV lead pacing threshold. Furthermore, the relationship between myocardial ischemia and a change in the pacing lead threshold might be supported by LV pacing failure at the multiple pacing vectors despite LV capture was achieved across a range of area at baseline. However, detection of myocardial ischemia was difficult, because the patient was asymptomatic for cardiac ischemia because of diabetes, and ST‐segment could not be evaluated during pacing rhythm. Although unipolar electrocardiograms recorded from the electrodes has been reported to be useful for demonstrating myocardial ischemia, the unipolar electrocardiograms of the LV lead were not recorded during the acute phase. In the present case, we initially detected the LV pacing failure from electrocardiographic findings, and then coronary angiography revealed a complete occlusion of the OM branch, perfusing the area around the implanted LV lead electrodes. After revascularization of the occluded coronary artery, the LV pacing lead threshold was improved.

In conclusion, myocardial ischemia was associated with a sudden rise in the pacing threshold after CRT‐D implantation. In case of a sudden rise in the pacing lead threshold, the presence of myocardial ischemia should be considered as one of the differential diagnosis.

